# Accounting for partiality in serial crystallography using ray-tracing principles

**DOI:** 10.1107/S1399004715011803

**Published:** 2015-08-25

**Authors:** Loes M. J. Kroon-Batenburg, Antoine M. M. Schreurs, Raimond B. G. Ravelli, Piet Gros

**Affiliations:** a Crystal and Structural Chemistry, Bijvoet Center for Biomolecular Research, Utrecht University, Padualaan 8, 3584 CH Utrecht, The Netherlands; b M4I Division of Nanoscopy, Maastricht University, PO Box 616, 6200 MD Maastricht, The Netherlands

**Keywords:** serial crystallography, *EVAL*, partiality of still data

## Abstract

Serial crystallography generates ‘still’ diffraction data sets that are composed of single diffraction images obtained from a large number of crystals arbitrarily oriented in the X-ray beam. Estimation of the reflection partialities, which accounts for the expected observed fractions of diffraction intensities, has so far been problematic. In this paper, a method is derived for modelling the partialities by making use of the ray-tracing diffraction-integration method *EVAL*. The method estimates partialities based on crystal mosaicity, beam divergence, wavelength dispersion, crystal size and the interference function, accounting for crystallite size. It is shown that modelling of each reflection by a distribution of interference-function weighted rays yields a ‘still’ Lorentz factor. Still data are compared with a conventional rotation data set collected from a single lysozyme crystal. Overall, the presented still integration method improves the data quality markedly. The *R* factor of the still data compared with the rotation data decreases from 26% using a Monte Carlo approach to 12% after applying the Lorentz correction, to 5.3% when estimating partialities by *EVAL* and finally to 4.7% after post-refinement. The merging *R*_int_ factor of the still data improves from 105 to 56% but remains high. This suggests that the accuracy of the model parameters could be further improved. However, with a multiplicity of around 40 and an *R*_int_ of ∼50% the merged still data approximate the quality of the rotation data. The presented integration method suitably accounts for the partiality of the observed intensities in still diffraction data, which is a critical step to improve data quality in serial crystallography.

## Abstract 2

Serial crystallography generates partial reflections from still diffraction images. Partialities are estimated with *EVAL* ray-tracing simulations, thereby improving merged reflection data to a similar quality as conventional rotation data.

## 1. Introduction   

X-ray free-electron lasers and high-brilliance undulator beamlines at synchrotrons have been used to perform serial (femtosecond) crystallography, collecting diffraction data from a large number (thousands up to millions) of micrometre-sized or nanometre-sized crystals (Chapman *et al.*, 2011 ▸; Boutet *et al.*, 2012 ▸; Redecke *et al.*, 2013 ▸; Gati *et al.*, 2014 ▸; Demirci *et al.*, 2013 ▸). Individual crystals may be hit by an X-ray pulse, thereby producing a diffraction pattern within the 10–50 fs pulse duration, before being vaporized by the transferred energy. This principle of ‘diffraction before destruction’ has been demonstrated by experiments on the Linac Coherent Light Source (LCLS) hard X-ray free-electron laser (Chapman *et al.*, 2011 ▸). Since the X-ray pulses are shorter than it takes for radiation-induced structural changes to occur, this approach of serial crystallography overcomes radiation damage, which has become a major problem with highly brilliant synchrotron sources (Weik *et al.*, 2000 ▸; Ravelli & McSweeney, 2000 ▸; Burmeister, 2000 ▸) using conventional rotation methods of collecting data from one or very few larger crystals. The diffraction images in serial crystallography are single snapshots of nonrotating crystals: so-called still images. As opposed to the conventional rotation data, the reflections are not fully integrated but are partials, except possibly when using future pink XFEL beams (Dejoie *et al.*, 2015 ▸). The particular orientation of the crystal lattice determines the extent of this partiality, which is a great unknown in the data-reduction process.

The specific challenges in data processing are the indexing of the stills, the reconstruction of full intensities and the merging of data obtained from different crystals, in addition to the handling of huge amounts of data. Three software packages are available to process serial X-ray diffraction patterns: *CrystFEL* (White *et al.*, 2012 ▸, 2013 ▸; White, 2014 ▸), *cctbx.xfel* from the *Computational Crystallographic Toolbox* (Sauter *et al.*, 2013 ▸; Hattne *et al.*, 2014 ▸) and *nXDS* (Kabsch, 2014 ▸). For indexing, rotation-method indexing packages such as *MOSFLM* (Leslie & Powell, 2007 ▸), *DirAx* (Duisenberg, 1992 ▸) and *LABELIT* (Sauter *et al.*, 2004 ▸) are being used. In 2010, Kirian and coworkers proposed a Monte Carlo integration method that, by averaging large numbers of diffraction spots, averages out the unknown partialities as well as differences in crystal size, beam flux and the incident spectrum (Kirian *et al.*, 2010 ▸). Thousands of diffraction images are needed for this method to converge (Boutet *et al.*, 2012 ▸). It is generally believed (White, 2014 ▸) that estimation of partialities could reduce the number of images needed for the Monte Carlo integration method and could improve the data quality. Three approaches have been proposed to estimate partialities. All three use post-refinement to improve the partiality correction factors and scale factors for each image. Kabsch (2014 ▸) derived an analytical expression for partiality from a Gaussian mosaic spread function. Comparison of ultrafine-sliced rotation images treated as stills or as normal rotation images gave satisfactory results. Kabsch includes a Lorentz factor for still data explicitly. The still data processing is not as good as one would expect, according to Kabsch. He concludes that this may be caused by two-dimensional rather than three-dimensional profile fits and the lack of other unimplemented corrections. White (2014 ▸) considers the overlap of reciprocal reflection volumes with a nest of Ewald spheres and calculates partialities from the distance of reciprocal-lattice points to the two limiting Ewald spheres. Using modelled data, White shows that the partiality estimation improve the data, with significant improvement of the statistics upon post-refinement. Most recently, Sauter (2015 ▸) and Uervirojnangkoorn *et al.* (2015 ▸) presented a partiality model that is implemented in *cctbx.xfel*. They calculated the intersection with the Ewald sphere of a spherical reciprocal-lattice point, where the radii of the lattice points are determined by mosaic spread and (asymmetric) beam divergence. Sauter (2015 ▸) also includes a parameter for the coherently scattering volume of mosaic blocks. Using this approach on XFEL data with post-refinement of crystal orientations, scale factors and beam parameters, the data are improved in quality as judged from molecular-replacement scores, structural refinement and anomalous difference maps (Uervirojnangkoorn *et al.*, 2015 ▸). Moreover, they show that reliable structures can be obtained with a lower number of images. Unfortunately, these authors do not mention merging *R* factors. Another correction that potentially improves Monte Carlo integration convergence for nanocrystals is explored by estimation of the crystal sizes and their corresponding diffraction power, as described by Qu *et al.* (2014 ▸). They show that the geometric correction factor, solely based on the maximum of the Laue interference function for each crystal with size *N_x_* × *N_y_* × *N_z_*, is superior to Monte Carlo integration for simulated data. Although the above efforts were made to improve processed serial crystallography still data, many questions still need to be addressed. Why do the data not improve rigorously with the current partiality-correction models? What factors exactly determine the partiality? Which errors dominate the partiality-estimation schemes?

Here, we describe an extension of the *EVAL* profile-prediction algorithm to process still images. *EVAL* is a data-reduction method designed for integrating reflection intensities through profile fitting using ray-tracing simulations (Duisenberg *et al.*, 2003 ▸; Schreurs *et al.*, 2010 ▸). We derived a general interference function that is valid for crystals of any size and effectively includes the shape transform. The diffraction process is simulated by typically 10 000 rays, which are diffracted by an equal amount of reciprocal-lattice vectors. In the rotation method, we bring reciprocal-lattice vectors onto the Ewald sphere by rotation around the spindle axis. However, in the still diffraction method we calculate the deviation from the exact Bragg condition for each ray and estimate its contribution to the total diffracted intensity using the interference function. By summation, the partiality of a reflection is obtained and, as we will show, also the still Lorentz factor. To test the approach, we used two still data sets collected on our in-house diffractometer using a single lysozyme crystal: one consisting of consecutive, stepwise stills and one consisting of stills from arbitrary orientations. Both were compared with conventional rotation data collected under the same conditions. We show that for these data sets the reflection partialities can be estimated by the ray-tracing simulation method and that the presented approach significantly improves the mean intensities of the observed reflections.

## 2. Diffraction theory   

**Figure 1:**
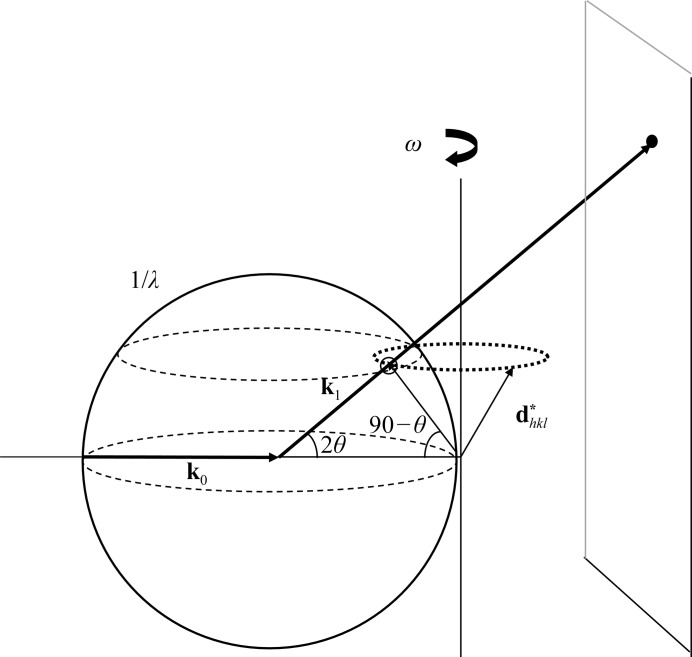
Ewald construction for a rotation experiment. An incident X-ray beam **k**_0_ with length 1/*λ* is reflected by a series of net planes in the direction **k**_1_ if the corresponding reciprocal-lattice vector 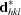 coincides with the Ewald sphere. In an arbitrary orientation of the crystal lattice 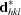 is not in a reflecting position but can be rotated around the spindle axis so that the Bragg condition is met and 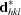 makes an angle of 90° − θ with the X-ray beam **k**_0_.

Reflection profiles from a crystal in *EVAL* are simulated by generating ray traces. We consider a crystal to be built up from small crystallites by dividing the crystal on a three-dimensional grid (sampled from a distribution **K**) that can have random orientations taken from a mosaic distribution (**M**). Incident X-rays are emitted from a virtual focus (*e.g.* a square area **F**) in direction **k**_0_ with respect to the crystallite and with wavelength λ (sampled from a spectral distribution **L**). A crystallite with an orientation of the reciprocal-lattice vector 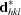 gives rise to a diffracted ray in direction **k**_1_ as determined by the Ewald construction. For **F**, **L**, **K** and **M** several statistical distributions are available (Schreurs *et al.*, 2010 ▸). In the simulation of rotation data the vectors 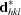 are rotated around the spindle axis so as to match the Bragg condition and then touch the Ewald sphere (Fig. 1 ▸).

In case of still diffraction experiments with one particular orientation of the crystal, none of the crystallite lattice vectors 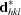 are exactly on the Ewald sphere. However, some vectors are within a certain tolerance in angular deviation (∊) of the Bragg angle θ and may give rise to diffracted intensity that is a function of ∊ (see below). The integrated intensity of all vectors 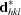 from the various crystallites depends on the mosaic spread, the wavelength dispersion, the beam size and divergence, the crystal size and the crystallite size itself. The latter corresponds to the coherently diffracting volume of the mosaic blocks, and the total reflected intensity of the crystal is the incoherent sum of all diffracted rays.

### 2.1. Still diffraction images   

The scattered intensity of a crystal at Bragg angle θ can be thought of as the coherent sum of scattering by *s* layers of thickness *d*, according to what we call the James–Buerger theory (James, 1958 ▸; Buerger, 1960 ▸). The scattered intensity of a single layer is
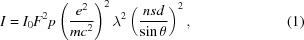
where *F* ^2^ is the squared structure factor, *I*_0_ is the incident photon flux, *p* is the polarization owing to the reflection, *e* ^2^/*mc* ^2^ is the Thomson scattering length of one electron and *n* is the number of unit cells per unit volume. A mosaic crystal is made up of tiny crystallites with associated reciprocal-lattice vectors 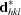 that are spread over an angular range μ, and each of them may not be perfectly oriented to be in Bragg condition. The *s* layers within such a crystallite then scatter slightly out of phase and their scattered intensity is given by the interference function
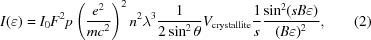
where ∊ is the deviation of the Bragg angle θ, *B* = 2π*d*cosθ/λ and *V*_crystallite_ is the reflecting volume of the crystallite.

**Figure 2:**
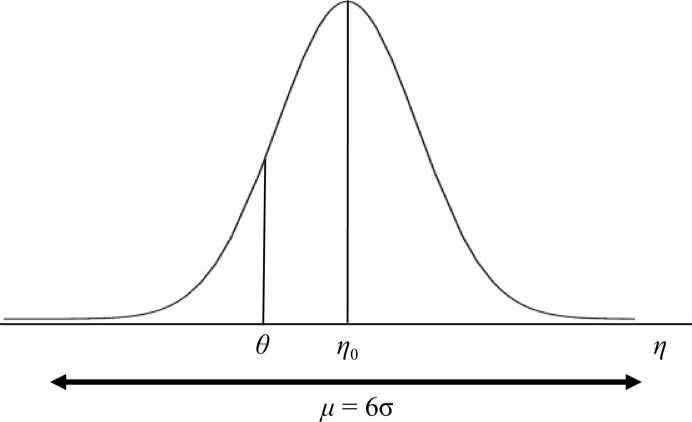
Angular spread of mosaic vectors. η_0_ denotes the angle of the central mosaic vector (*i.e.* that which makes an angle of 90° − η_0_ with the X-ray beam), which may be deviating from the ideal Bragg angle θ. The full mosaic spread is defined as six times the standard deviation of a Gaussian distribution.

The James–Buerger theory can be extended by writing the total diffracted intensity as an integral over all possible orientations of the crystallite vectors 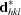 that make angles of 90° − η with the incident X-ray beam (90° − θ in the Bragg condition) and replacing *V*_crystallite_ by the volume of the crystal *V* and ∊ by θ − η (see Fig. 2 ▸). This results in
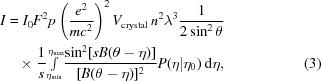
where *P*(η|η_0_) is the probability distribution of η angles given the angle η_0_ of the central reciprocal-lattice vector 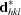
(0) of the crystal as obtained from the unit-cell matrix.

The mosaic spread, the divergence of incident rays, the wavelength variations and the crystallite positions being slightly off-centre in a larger crystal are the cause of deviations ∊ for the individual rays. For the discussion here, we will concentrate on the mosaic spread, but the other parameters are accounted for as well in our ray-tracing simulations.

The distribution function can take several forms. Suppose that *P*(η|η_0_) is uniform, while 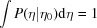, then the integral over dη in (3) reduces to *s*π/*B* and 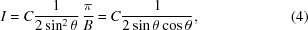
where *C* = *I*_0_ *F* ^2^ *p*(*e* ^2^/*mc* ^2^)^2^λ^3^ *n* ^2^. The last term in (4) is familiar: it is the Lorentz factor for rotation in the equatorial plane and is equal to the powder Lorentz factor. Thus, for still images the powder Lorentz factor applies. When *P*(η|η_0_) is a normal or an otherwise monotonous distribution, we should explicitly include it in the calculation of (3). However, it cannot be reduced to a simple trigonometric function nor to an erf (see Kabsch, 2014 ▸) because of the presence of the sinc function in the integral. Instead, it can be evaluated numerically.

### 2.2. Rotation diffraction images   

Rotation images can be regarded as a superposition of many stills separated by an infinitesimal rotation angle ω. The integrated intensity for these is
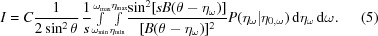
In a sufficiently large ω scan each 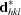 vector makes a complete pass through the Ewald sphere, so that we can write
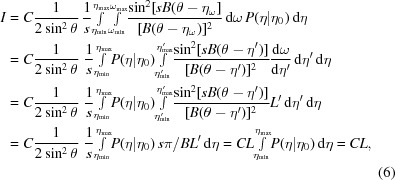
where *L*′ is the duration component of the Lorentz factor *L* for the rotation experiment (equal to the reflection range expansion factor in Kabsch, 2014 ▸). The rotation Lorentz factor may alternatively be written as 1/[**d** ^*^(**k**_0_ × **ω**)] (Milch & Minor, 1974 ▸). In case of rotation in the equator, (6) reduces to (4). In rotation data, therefore, the specific distribution function *P*(η) is irrelevant to the integrated intensity and complete reflections are obtained.

### 2.3. Implementation of the interference function in *EVAL*   

In *EVAL* a large number of vectors 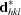
(*i*) are generated from a Gaussian or Lorentzian two-dimensional mosaic distributions (**M**) and combined with vectors **k**_0_ from **F**, **L**, **K** distributions. The contribution of each of these to the scattered intensity is calculated with
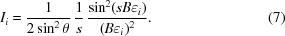
Summing all contributions gives the total scattered normalized intensity (*i.e.* *C* = 1.0; see text below equation 4), which is effectively an integral over d*η*, d**k**_0_ and, to a minor extent, d*λ*, because our beam is almost monochromatic. This normalized intensity is stored in the parameter ‘partiality’ after correction for the still Lorentz factor, *i.e.* the partiality is 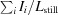. The only new parameter introduced is the number of unit cells in the crystallite *N*_cell_, where *s* = *N*_cell_(|*h*| + |*k*| + |*l*|), the number of reflecting planes, while the crystallite size equals *sd*_*hkl*_.

Every 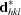
(*i*) produces its own impact on detector pixel coordinates (*x*, *y*) and is weighted by contribution *I_i_*. All impacts together build the two-dimensional reflection profile that is used as the model profile in the *EVAL* least-squares fit to obtain the observed integrated intensity for each diffraction spot on an image. Both the observed intensity and the summed interference function (7) contain the still Lorentz factor, and by dividing one by the other we extract *F* ^2^. We also correct for the polarization and apply possible incidence corrections.

### 2.4. Laue interference function   

In this paper, we follow the James and Buerger approach, as explained in §[Sec sec2.1]2.1, for deriving diffracted intensities by crystals. The resulting interference function only depends on the deviation ∊ from the Bragg angle θ and the number of unit cells contained in the crystal. An alternative is to use the three Laue conditions, and the squared sinc function in (3) is replaced by
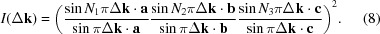
Here,Δ**k** = **k**_1_ − **k**_0_ and *N*_1_, *N*_2_ and *N*_3_ are the number of unit cells in the three periodic axis directions. In Appendix *A* [App appa], we show that the two approaches are exactly the same.

(8) is often referred to as the shape transform of the crystal (Kirian *et al.*, 2010 ▸; Spence *et al.*, 2011 ▸).

### 2.5. Impact positions and refinement   

Peak-position refinement in *PEAKREF* (Schreurs, 1999 ▸) minimizes the peak-position residuals and the Bragg angle deviation ∊_0_ of the central reciprocal-lattice vector either using peak maxima from the peak search or using optimized profile centroids from the *EVAL* profile fit. Inclusion of ∊_0_ in the unit-cell matrix refinement avoids divergence of unit-cell orientations through rotations perpendicular to the incident beam, as discussed by Sauter *et al.* (2014 ▸). Similarly, Kabsch (2014 ▸) uses the angular deviation τ divided by the mosaic spread σ_M_ in the target function for peak refinement. All three approaches use the β axis, defined for each reflection as the axis perpendicular to the incident and diffracted beams, to calculate the deviation from the Bragg angle θ (Schutt & Winkler, 1977 ▸). For each still image, the following target function was minimized to refine the unit-cell matrix, 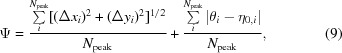
where Δ*x*_*i*_ and Δ*y*_*i*_ are the differences of observed and calculated peak positions. *PEAKREF* can optimize many instrumental parameters such as detector-offset positions, primary beam direction and crystal position, which in the current analysis were fixed in the still data refinement and based on the rotation data (see below).

**Figure 3:**
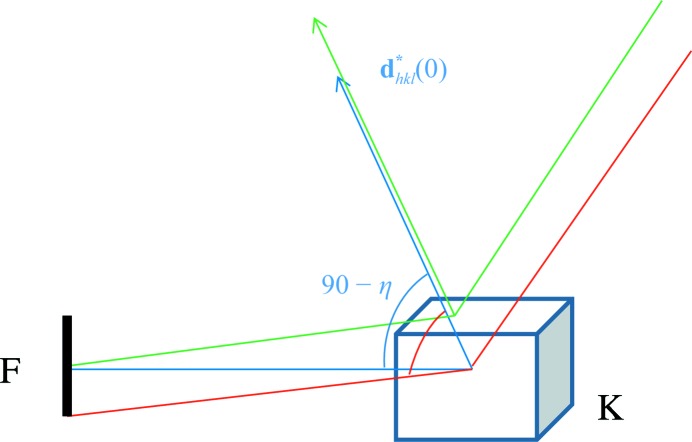
Schematic illustration of conditions causing an apparent shift in 2θ. A mosaic vector 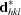
(0) is slightly off the Bragg condition for an X-ray beam from the centre of the focus **F** (blue line) and makes an angle of 90° − η with the X-ray beam. However, the Bragg condition tends to be fulfilled with rays emerging from the lower part of the focus (red line; the arc corresponds to an angle of 90° − θ) or for the upper part of the crystal (green line). Therefore, the reflected beam appears at a larger 2θ angle. Owing to averaging of many rays this effect is not, or is hardly, visible for rotation images, but it is for still images or ultrafine-sliced images. As the shift in apparent 2θ is always in the direction of 2η, we can account for this by the ‘flex’ parameter in *PEAKREF*.

We found that the peak-position residuals from the post-refinement were much smaller than from the peak maxima found on a single still image, despite the much larger number of peaks. This was caused by a shift in the observed θ value for the partial reflections with large ∊_0_. For large mosaic crystals such as our lysozyme crystal measured with a divergent beam, these shifts in θ occur because only distinct directions of the primary rays or distinct points on the crystal are active dependent on the deviation ∊_0_ (Fig. 3 ▸). A negative value for ∊_0_ results in an apparent larger θ and a positive ∊_0_ in an apparent smaller θ. This θ-divergence effect has to be taken into account when the cell matrix is determined and refined from peak positions. We introduced a parameter ‘flex’ in *PEAKREF* that is jointly refined and takes account of this shift. The ‘flex’ parameter turned out to have a constant value for all still images and it appears to be a property typical for the crystal and the beam divergence of the particular experiment.

### 2.6. Post-refinement   

We implemented a post-refinement procedure in which both the peak positions from the *EVAL* integration and the partialities could be refined. For this purpose, we calculate the mean intensity of all equivalent reflections *h* as 
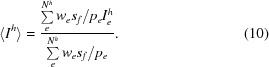
Weights are obtained from the standard deviations from the *EVAL* profile fit (Schreurs *et al.*, 2010 ▸) and are given by *w*_*e*_ = 1/σ_*e*_^2^. The partialities *p_e_* arise from the *EVAL* ray-tracing simulation, and the image scale factors *s_f_* are determined in *ANY* (Schreurs, 2007 ▸), assuming a constant sum of Bragg intensities in each frame. The summation in (10) runs over all equivalents of reflection *h* (*N^h^*) in the data set. In *PEAKREF* image scale factors *s_f_*′ and unit-cell parameters and crystal orientation angles are refined using the target function
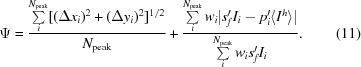
We specifically include peak positions in this refinement step in order to avoid unwanted divergence from peak position-derived unit-cell parameters and orientations. In this post-refinement step the *EVAL* ray-tracing procedure is not repeated to obtain partialities; instead, we use a fitted partiality *versus* ∊ curve with a single Gaussian. The parameters in the Gaussian were kept fixed in the refinement; ∊ changes with the unit-cell parameters from which we recalculate the partiality (*p_i_′*).

## 3. Materials and methods   

### 3.1. Crystal preparation   

Hen egg-white lysozyme (Sigma–Aldrich, Schnelldorf, Germany) was crystallized using the hanging-drop vapour-diffusion method with a protein concentration of 75 mg ml^−1^ in 0.1 *M* sodium acetate buffer pH 4.8. The precipitant consisted of 0.1 *M* sodium acetate buffer pH 4.8, 10–15%(*w*/*v*) sodium chloride, 30%(*v*/*v*) ethylene glycol (Sutton *et al.*, 2013 ▸). Drops of 4 µl were set up with a 1:1 protein:precipitant ratio.

### 3.2. Data collection   

A crystal of dimensions 250 × 250 × 150 µm was vitrified in a cold N_2_-gas stream from an Oxford Instruments 700 series jet operated at 100 K. Data were collected on a Bruker–AXS X8 Proteum in-house source with Cu *K*α radiation. The rotating anode was operated at 45 kV and 60 mA. The reference rotation data set was collected by rotating over 190° in φ in 0.5° steps per frame. Data were recorded on a PLATINUM^135^ CCD detector with a sample-to-crystal distance of 52 mm. 380 still images were collected with identical angular settings as the starting angles for each of the rotation frames; thus, 380 still images were collected at 0.5° intervals. An additional 394 stills were recorded by random selections from ω scans 0–7° in ω apart at 15 different ω, κ and φ goniometer settings. The exposure time for all images was 5 s.

### 3.3. Data processing and analysis   

*VIEW* was used for image display and peak search (Schreurs, 1998 ▸). Both the rotation images and stills were indexed using *DirAx* (Duisenberg, 1992 ▸). Almost all stills could be indexed without manual intervention. Bravais lattice constraints were applied and the unit cells were made congruent (using the goniometer positions), ensuring a consistent choice of unit-cell axes. The unit-cell matrix and detector positions were refined from 649 peak positions in the rotation data. For the still peak positions we used different refinement options. In the first approach we made use of our knowledge of the relative positions of the goniometer axes, so that a global single unit-cell matrix could be refined against 10 728 peak positions. In the second approach, we determined and refined a unit-cell matrix for each image from 300 peak positions, as would be the normal procedure in serial crystallography. The detector-offset positions were taken from the peak-position refinement of the rotation data. The unit-cell matrix was refined against the observed peak positions, using the ‘flex’ parameter to account for apparent shifts in θ, simultaneously minimizing the off-Bragg angle ∊_0_ (9). Using the unit-cell matrix, we extracted three-dimensional and two-dimensional reflection boxes for rotation and still images, respectively, and processed these with *EVAL*. For every reflection, 10 000 rays were simulated and the impacts were collected in pixels contained in the box. In case of still data every individual ray is associated with a reciprocal-lattice vector 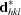
(*i*) with a small angular deviation from the Ewald sphere ∊_*i*_ and is weighted by the interference function (7). The impact position on the detector is given by the direction of the shortest distance of the reciprocal-lattice vector to the Ewald sphere. The divergence effects are accounted for in the ray tracing and thus the profiles are generated correctly at deviating positions in θ (*i.e.* without the need for a ‘flex’ parameter as used at the peak-refinement stage).

The parameters for crystal size, mosaic spread and beam divergence were optimized automatically in the reflection profile fitted to ∼50 reflections with *I*/σ(*I*) > 20 using a simplex method (see Schreurs *et al.*, 2010 ▸). For comparison reasons, identical values of parameters in the ray-tracing simulations were used for both types of data sets, although a similar optimization can be performed for still images. In addition, for the still images we used *N*_cell_ = 25 in the interference function (the number of unit cells in a crystallite as described in §[Sec sec2.3]2.3). Sampling of the interference function converges much faster with low values of *N*_cell_, typical for nano-sized crystals. The current data imply a larger value of *N*_cell_, which in the current implementation would require many more rays (up to 10^6^ instead of 10 000) to sample reflection profiles smoothly. The integrated intensities are obtained by a least-squares fit of the three-dimensional and two-dimensional model profiles to the observed pixel intensities for the rotation and still reflections, respectively. *EVAL* then delivers the profile-fitted, Lorentz- and polarization-corrected intensity values in an XML-type datafile that is further processed in *ANY* (Schreurs, 2007 ▸). In this program, we determine image scale factors, correct for the partiality factor and output the intensities and standard deviations to an hkl- or mtz-type file. Many of the graphical plots and statistical analyses are made using *ANY*.

All still images were also processed with the *CrystFEL* software suite v.0.5.1 (White *et al.*, 2012 ▸). Structural refinements were carried out with *REFMAC*5 (Murshudov *et al.*, 2011 ▸) and scaling between data sets with *SCALEIT* (Howell & Smith, 1992 ▸), both from the *CCP*4 suite (Winn *et al.*, 2011 ▸). *ANODE* (Thorn & Sheldrick, 2011 ▸) was used to calculate anomalous difference densities.

## 4. Results   

We collected rotation and still diffraction data from one lysozyme crystal and formed three data sets for analysis: a 190° rotation data set collected in 380 images in ranges of 0.5° for reference, a consecutive still data set of 380 images collected in steps of 0.5°, and this consecutive data set combined with 15 wedges of separate arbitrary orientations totalling 774 images (Table 1 ▸). Indexing and peak refinement by *PEAKREF*/*EVAL* and *CrystFEL* yielded unit-cell dimensions that varied by ∼0.3–0.7% between the separate stills. Initially, the average residuals in peak positions for the still data were significantly larger than for the rotation data. Introduction of the ‘flex’ parameter, which takes into account the apparent shift in θ owing to divergence effects (see §[Sec sec2.4]2.5), reduced the positional residuals of peak maxima on still images significantly: from 0.13 to 0.07–0.08 mm on average. This deviation is only slightly larger than that observed for the rotation data, which was 0.06 mm. The residual in rotation angle for the rotation data was 0.039° (for 0.5° scan width). For the still data the deviation from the Bragg angle ∊_0_ was 0.18°, which is consistent with a mosaic spread of 0.5°. Relaxing the unit cells by a separate matrix for each image lowered the ∊_0_ residuals to 0.14° (compare ‘single unit cell’ *versus* ‘unit cell per image’ in Table 1 ▸). In our setup the orientation of each matrix was known because the crystal orientation was set using a gonio­meter. The r.m.s. deviations between the set and refined orientations of the reciprocal-lattice vectors a*, b* and c* were 0.03° for the consecutive still data and increased to ∼0.10° using all still data (consecutive and random orientations). Overall, the number of observations taken into account by *EVAL* were 106 × 10^3^ for the rotation data set, 325 × 10^3^ for the consecutive still data and 657 × 10^3^ for all still data, whereas *CrystFEL* took 733 × 10^3^ into account for all still data (Table 2 ▸). All processed sets resulted in ∼8300 unique reflections. The multiplicity of the consecutive still data was roughly three times that of the rotation data, indicating that reflections were, on average, sliced through three times in our still data-collection experiment.

The statistics for the integration and merging of data for the rotation and still data are shown in Table 2 ▸. Processing of the reference rotation data yielded an internal merging *R*_int_ of 3.8% with an 〈*I*/σ(*I*)〉 after merging of 47.7. Processing of the still diffraction data without correction, referred to as Monte Carlo averaging, produced *R*_int_ values exceeding 100% and 〈*I*/σ(*I*)〉 values that were about fourfold lower than that for the rotation data using the same number of images. Application of the still Lorentz correction (4) slightly increased the *R*_int_ (Table 2 ▸).

**Figure 4:**
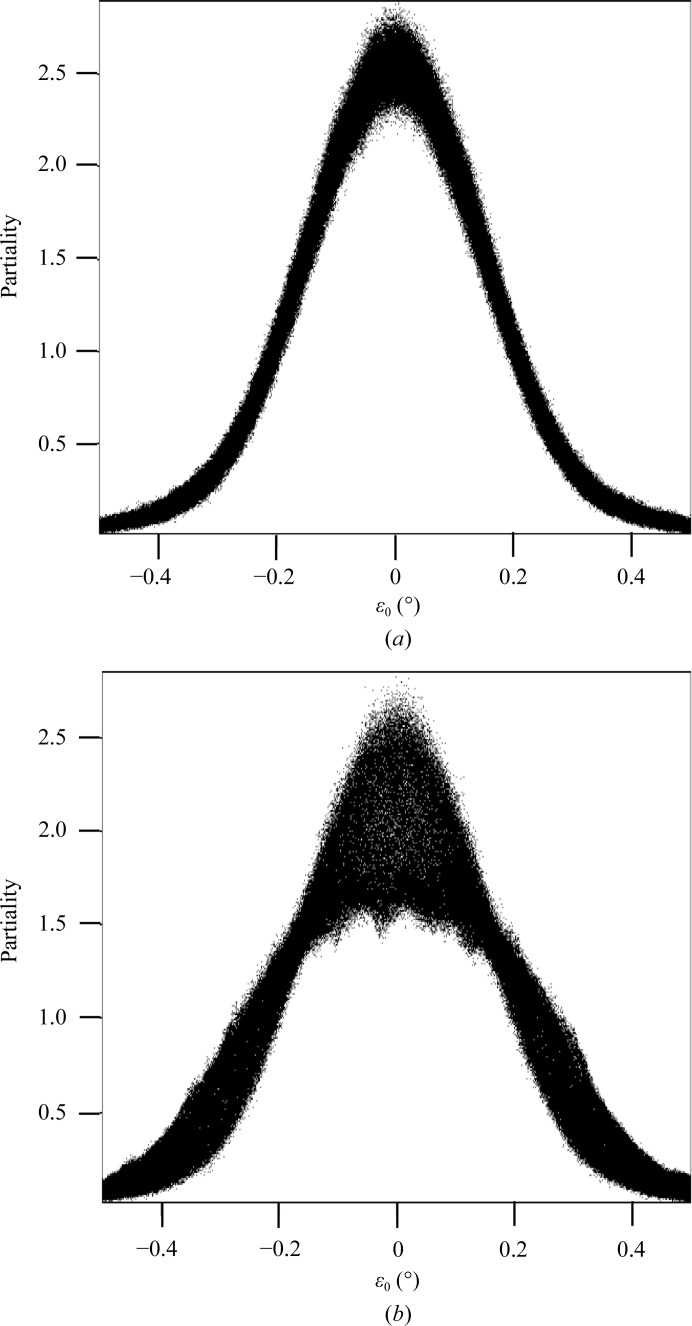
Partialities of reflections plotted against their ∊_0_. (*a*) From the ray-tracing model used in this paper, (*b*) from ray tracing using a long focus.

**Figure 5:**
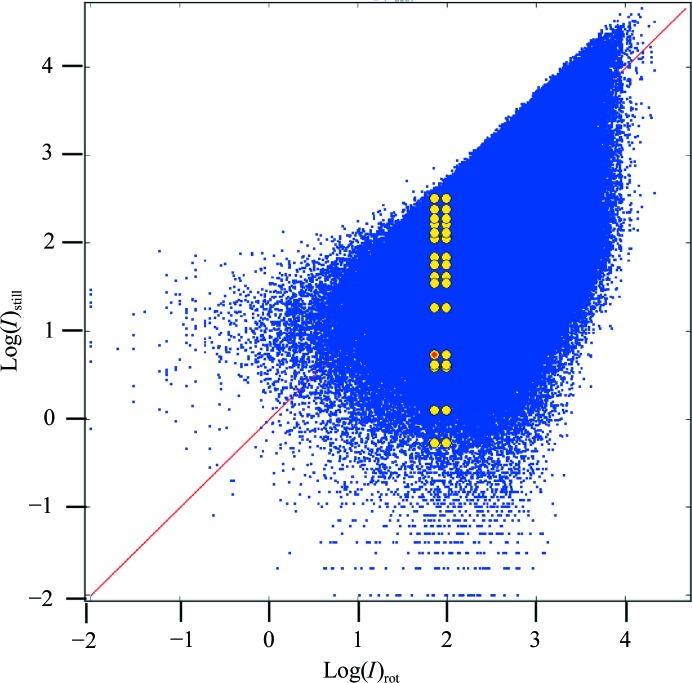
Lorentz-corrected still (not partiality-corrected) *versus* Lorentz-corrected rotation intensities. All occurrences of reflection 1, −6, 5 are marked by yellow dots. No scaling was applied so that intensities are on an absolute scale. Dots above the red line with slope = 1.0 indicate that the observed still intensity is larger than its corresponding rotation intensity, resulting in a partiality larger than 1.0. On average, the equivalent observations of a unique still reflection are equal to the rotation intensity (see text).

**Figure 6:**
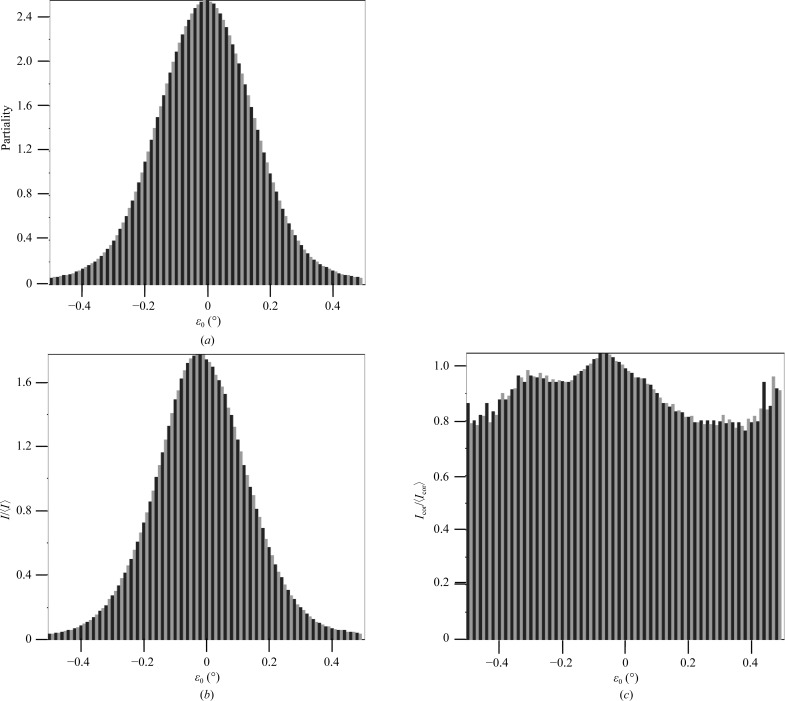
Histogram of reflection data *versus* ∊_0_. (*a*) Partialities, (*b*) *I*/〈*I*〉 for equivalent reflections and (*c*) *I*/〈*I*〉 after partiality correction.

To estimate partialities, we determined the parameters for mosaic spread, divergence of the incident beam, crystal size and *N*_cell_ by optimizing two-dimensional profile fits using figures of merit (Schreurs *et al.*, 2010 ▸) on a subset of reflections in *EVAL*. Mosaic spread was set to 0.5°, beam divergence to 8.6 mrad, crystal size to 130 × 130 × 130 µm (although we estimated a slightly larger size when selecting the crystal under the microscope) and *N*_cell_ to 25. The ray-tracing procedure yielded partialities which showed a Gaussian-like distribution with ∊_0_ (Fig. 4 ▸ *a*). Notably, the computed still partialities are not normalized and exceed a value of 1, and hence are used as relative scale factors. In rotation data the partiality is defined up to 1 for a fully observed reflection (Rossmann & Beek, 1999 ▸); in contrast, the partiality in still diffraction is determined by the angular width of the intersection with the Ewald sphere, which depends on various instrumental and crystal parameters such as those given by (3). Lorentz-corrected still and (Lorentz-corrected) rotation reflections on average give the same absolute intensities. Fig. 5 ▸ shows that some still partialities are larger than 1.0 and the still intensities scatter around the rotation intensity. Further, to illustrate that the partialities depend strongly on the precise ray-tracing model parameters, Fig. 4 ▸(*b*) shows the partialities as a function of ∊_0_ in the case of a long focus for the incident beam, which results in two Gaussian-like curves superimposed. This implies that a simple Gaussian model for the partiality is not always correct. When divided into ∊_0_ bins, the observed average intensities correlate well with the estimated partialities (Fig. 6 ▸). Application of the partiality model resulted in average *I*/〈*I*〉 values that varied around the ideal value of 1.0. Subsequent merging of these data, *i.e.* with both Lorentz and partiality corrections applied, reduced the *R*_int_ values to 57 and 63% for the data sets with consecutive and all stills, respectively.

**Figure 7:**
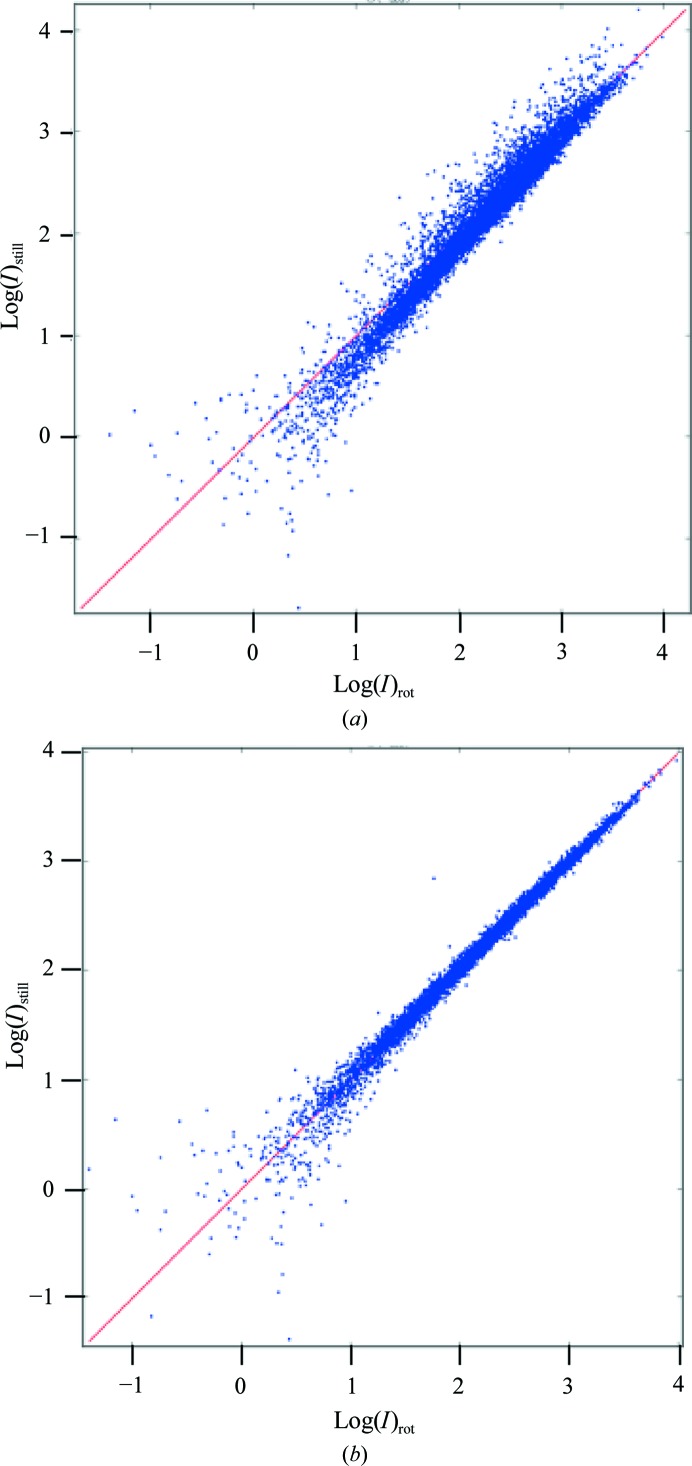
Scatter plots of still *versus* rotation reflection intensities. (*a*) Uncorrected Monte Carlo and (*b*) corrected reflection intensities after merging in point group 4/*mmm*.

Next, the effects of Lorentz and partiality correction were evaluated by comparing the data with the reference rotation data set. The uncorrected and the Lorentz-corrected intensities have high internal *R*_int_ values of 104.9 and 106.5%, respectively, consistent with the scattering in Fig. 5 ▸. The Lorentz- and partiality-corrected intensities have an *R*_int_ of 63.8%. Upon merging the data to unique reflections the agreement with the rotation data improved dramatically; the scatter diagrams in Fig. 7 ▸(*a*) and 7 ▸(*b*) reflect the improvement corresponding to the uncorrected (Monte Carlo) and corrected (Lorentz and partiality) data. The effects from the still data corrections are more clearly demonstrated by the *R* factors with respect to the reference rotation data, which we refer to as *R*_comp_ (Table 3 ▸). *R*_comp_ (on intensities) was 26% using Monte Carlo averaging. Application of the Lorentz correction alone decreased the *R*_comp_ to 12%. Application of both Lorentz and partiality corrections yielded an *R*_comp_ of 5.3%.

**Figure 8:**
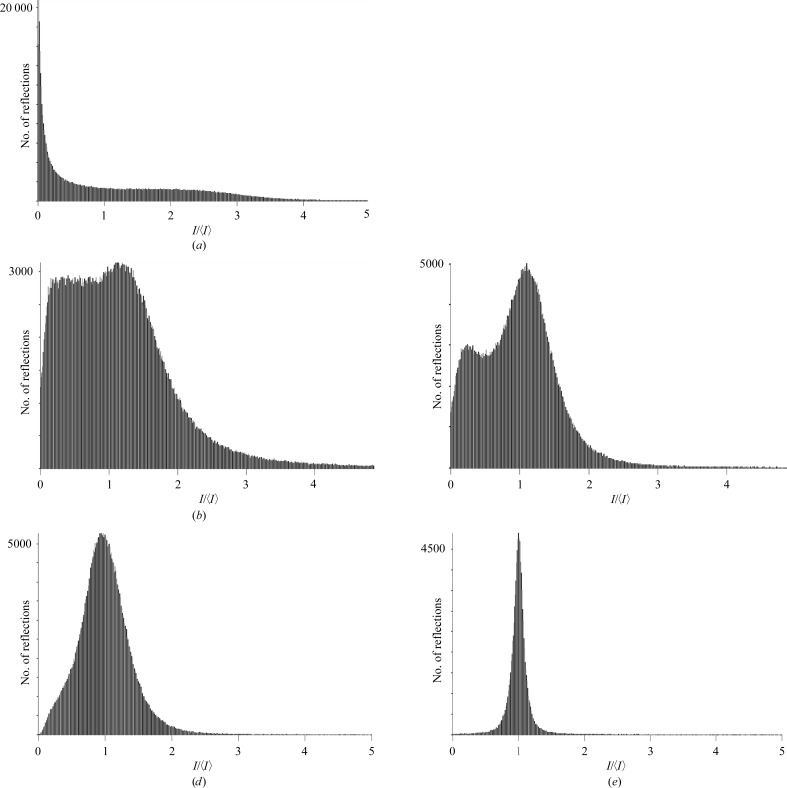
Histogram of *I*(*hkl*)/〈*I*(*hkl*)〉. (*a*) Uncorrected, (*b*) partiality-corrected, (*c*) partiality and post-refined still data, (*d*) the same as (*c*) but omitting weak reflections and (*e*) rotation data

Although the Lorentz and partiality corrections significantly improved the quality of the merged data, the merging *R*_int_ value remained high (*i.e.* 63.8% for all still data). To improve the partialities, we performed post-refinement of the image scale factor, unit-cell parameters and orientations, minimizing the target function of (11). Post-refinement of the ‘all stills’ data gave scale factors of 0.84–1.35 (additional to the scale factor *s*_*f*_ used in equation 10) and sharpened the distribution of unit-cell dimensions, with virtually no effect on the variation of crystal orientations (Table 1 ▸). These adjustments resulted in a significant, but modest, reduction of *R*_int_ from 63.8 to 55.7% (Table 2 ▸). The progress in the precision of processing the data is reflected by the distributions *I*(*hkl*)/〈*I*(*hkl*)〉 shown in Fig. 8 ▸. Ideally, *I*(*hkl*)/〈*I*(*hkl*)〉 values form a sharp distribution around 1 (as a reference, we depict the distribution resulting from the rotation data in Fig. 8 ▸ *e*). Figs. 8 ▸(*b*) and 8(*c*) reflect the striking improvement obtained by modelling the partiality in *EVAL* and subsequent post-refinement. Fig. 8 ▸(*d*) shows that mainly the weak data do not profit from the post-refinement. Comparison of the merged data sets shows that the improvement in precision is matched by an improvement in accuracy. Post-refinement reduced the *R*_comp_ from 5.3 to 4.7% (Table 3 ▸).

To illustrate the data quality, we refined the lysozyme crystal structure and computed anomalous difference densities. The structure was refined starting from PDB entry 193l (Vaney *et al.*, 1996 ▸) against the reflection data using *REFMAC*, and we observed similar *R*_work_ and *R*_free_ values for the differently processed data (Table 4 ▸). Significant differences between the methods were observed for the resulting average isotropic *B* factors. Monte Carlo averaging of the data in *CrystFEL* and *EVAL* yielded increased *B* factors (21–25 Å^2^) compared with the reference defined by the rotation data set (〈*B*〉 = 13.8 Å^2^). The Lorentz correction had a large effect on the *B* factors and produced an average *B* factor of 11.8 Å^2^; this large effect on the *B* factors is explained by a comparable fall-off in θ of the Lorentz factor and the temperature factor. When the Lorentz and partiality corrections were both applied, the *B* factors became more similar to those obtained when using the rotation data (13.2 *versus* 13.8 Å^2^). Anomalous differences are much more sensitive to the accuracy of the data than structure refinement. We generated anomalous difference densities based on the processed data sets using phases from the refined structure by *ANODE*. For the methionine sulfur positions the anomalous density from the rotation data gave a peak height of 13.3σ. The uncorrected, Monte Carlo averaged still data yielded a weak anomalous signal: a 4.2σ peak for methionine S, corresponding to 32% of the peak height using the rotation data. Lorentz correction improved the methionine S signal to 35%, whereas including partiality corrections resulted in 47% of the signal. Finally, this signal improved to 54% after post-refinement. This shows that both Lorentz and partiality correction improved the intensities deduced from the still data.

We tested the effect of data-set size by limiting the still data to 60 images (Table 5 ▸). For the reduced ‘consecutive still’ data we used images 250–310. For the ‘random still’ data 60 images from three different wedges were used. For these limited data sets (91.7 and 97.3% completeness, respectively), the *R*_free_ factors show that the structure quality deteriorated. Furthermore, the anomalous signal is largely lost. For both structure refinement and anomalous density analyses the Lorentz and partiality-corrected data outperform the noncorrected Monte Carlo processed data.

## 5. Discussion and conclusions   

We used our ray-tracing profile-prediction methods to model partialities of the observed reflections in still diffraction data and adapted the programs *PEAKREF* and *EVAL* to process still diffraction images. By taking experimental conditions into account, we compute 10 000 rays generated from focus, crystal grid points, wavelength spectrum and mosaic distributions, and calculate the interference-function weighted contribution to an observed reflection and hence derive its partiality. Our formalism implicitly models for the Lorentz factor, mimicking the contribution of the Lorentz factor to the observed intensities. Our approach differs fundamentally from other still data-processing methods. Kabsch (2014 ▸) defined an analytical erf function for the partiality, which is the integral over a Gaussian mosaic function. It is equivalent to our integral in (3) for an infinitely sharp sinc function (implying that integration over this function is complete within a solid angle smaller than the pixel size of the detector), while ignoring broadening effects other than the mosaic spread. Kabsch explicitly corrects for the still Lorentz factor. White (2014 ▸) and Sauter (2015 ▸) use reciprocal-lattice point volumes for calculating the partiality. White (2014 ▸) accounts for spectral width and beam divergence by calculating the overlap of a reciprocal-lattice volume with a nest of Ewald spheres. Sauter (2015 ▸) and Uervirojnangkoorn *et al.* (2015 ▸) use a single Ewald sphere and calculate the intersection with a spherical reciprocal-lattice volume, the size of which is determined by beam divergence, mosaic spread and spectral dispersion. Both approaches account for increase of reciprocal diffracting volume with resolution, and in this way for the wider range of acceptable off-Bragg angles (d∊; see Appendix *A* [App appa]). However, both approaches lack the reflectivity part of the Lorentz factor (dΩ; see Appendix *A* [App appa]). If the spectral width of the beam becomes large, an additional Lorentz factor needs to be accounted for, as used in the Laue method (Zachariasen, 1945 ▸). Uerviroj­nangkoorn *et al.* (2015 ▸) very recently presented their results on XFEL data. They showed that the *R*_work_ and *R*_free_ of refined structures improved and part of the anomalous signal was retrieved. Unfortunately, they do not provide merging *R*_int_ or a comparison to a rotation data set, *i.e.* *R*_comp_, to evaluate the resulting quality of the data more directly. In our approach, the integration of (3) is achieved by simulation of the rays that contribute to an observed reflection spot. Because of the simulation, the derivation of analytical functions for the various effects is not needed and the Lorentz effect is implicitly taken into account. Moreover, the interference function can be taken into account in our approach.

For the initial development of the method, we used an experimental setup that allowed a direct comparison to the conventional rotation method. Our analysis showed a dramatic improvement in data quality after partiality and Lorentz correction. Both data processing and structure refinement showed that Lorentz correction is important and that omission of the Lorentz correction strongly affects the temperature factor. The anomalous sulfur densities increased 1.7-fold upon Lorentz and partiality correction of the still data. Overall, our approach markedly improved the *R*_comp_ factor between the intensities derived from rotation and still data from 26% to a final value of 4.7% after Lorentz and partiality correction and post-refinement.

Concurrent with the improvement in the final data quality upon Lorentz and partiality correction in *EVAL*, the internal merging *R*_int_ decreased from 105 to 64%. When we were developing the method, we hoped that post-refinement of the parameters would improve the final unique intensity data as well as further reduce the internal merging *R*_int_ factor. Post-refinement improved the precision of the modelled unit-cell dimensions and scale factor per image, although the error in modelled crystal orientations remained ∼0.1°. These more precise parameters indeed improved the resulting intensities (*R*_comp_ decreased from 5.3 to 4.7%). The internal statistics improved as well (*R*_int_ decreased from 64 to 56%); however, the final *R*_int_ factor remained high. This high *R*_int_ could be owing to features that were not included in our ray-tracing model, such as possible asymmetry in the focus, (anisotropic) mosaic spread or crystal form, or absorption by the crystal. Notably, crystal absorption may have a significant effect on the presented data because a relatively large crystal was used in this experiment. Crystal absorption is likely to be negligible when data are collected from microcrystals or nanocrystals, as is the case in serial crystallography. Obviously, further development of our approach is needed to account for the experimental conditions of serial (femtosecond) crystallography using XFEL or synchrotron sources. Automated schemes will be needed to model, for example, the large number of single-crystal diffraction images and fluctuations in beam spectra. In general, comprehensive modelling of the relevant experimental conditions should improve both the internal merging statistics and the resulting intensities. Not modelling significant effects that are present in the data can only be overcome by collecting more data to allow the averaging out of these effects by the Monte Carlo approach. In a real-case scenario the rotation data will not be available to evaluate the data quality, and an *R*_int_ of ∼50% may possibly be a practical metric to judge the resulting data quality.

Overall, we have shown that ray tracing can produce reliable partialities that improve the resulting data quality originating from still diffraction images. Moreover, our method is versatile and allows the modelling of a wide variety of effects, including those that yield non-Gaussian, asymmetric effects on the diffraction spot. In particular, the approach can take the interference function into account, which will be critical for processing data obtained from nanocrystals. Thus, in this paper we have presented the theoretical framework and demonstrated the potential of the ray-tracing methodology for processing still diffraction data.

The rotation and still diffraction images are available at http://rawdata.chem.uu.nl/c003.

## Figures and Tables

**Table 1:** Indexing and peak-refinement statistics

			*EVAL*, consecutive stills		*EVAL*, all stills	
	*EVAL*, rotation	*CrystFEL*, all stills	Single unit cell	Unit cell per image		Post-refined
No. of images	380	774	380	380	774	
No. of peaks used for indexing	649	300490†	10728	259300†	289300†	
No. of peaks used in refinement	101246		217272	113418	230759	396201
Unit-cell parameters						
*a*, *b* ()	78.666	78.73579.453	78.834	78.76579.041	78.67979.119	78.71979.049
*c* ()	36.819	36.81937.481	36.842	36.70636.906	36.70636.989	36.81036.980
Orientation r.m.s. ()						
a*				0.028	0.102	0.103
b*				0.038	0.108	0.095
c*				0.035	0.107	0.111
Average residuals						
Horizontal, vertical (mm)	0.0622		0.0884	0.0785	0.0766	0.099
_0_ ()	0.0390		0.1850‡	0.1474‡	0.1496‡	0.173‡

† Per image.

‡ Average angular deviation of central reciprocal-lattice vector d* with Ewald sphere (see text for explanation).

**Table 2:** Statistics for the integration and merging of the rotation and still data

			*EVAL*, Monte Carlo		*EVAL*, Lorentz corrected		*EVAL*, partiality corrected		
	*EVAL*, rotation	*CrystFEL*, all stills	Consecutive stills	All stills	Consecutive stills	All stills	Consecutive stills		All stills
Unit cell			Image	Image	Image	Image	Single	Image	Image
No. of reflections	106021	733504					320139	325753	657782
No. unique†	8291						8301	8328	8352
Multiplicity	12.8	88.4					38.6	39.1	78.8
Completeness (%)	100	100					100	99.0	99.2
CC_1/2 _(%)	100		87.4 (80.3)	94.1 (88.6)	88.6 (79.9)	94.3 (88.5)	97.3 (90.9)	95.3 (88.1)	97.5 (90.9)
*I*/(*I*)	13.9 (1.7)		‡	‡	‡	‡	‡	‡	‡
*I*/(*I*), merged	47.7 (20.3)	10.1 (5.7)	8.0 (5.1)	11.5 (7.4)	8.1 (5.4)	11.8 (7.7)	12.0 (7.4)	11.0 (6.6)	15.1 (9.0)
*R*_int_ § (%)	3.8 (7.1)		106.2 (94.5)	104.9 (91.2)	108.0 (97.1)	106.5 (94.4)	57.5 (58.1)	62.6 (62.6)	63.8 (63.4)
Post-refinement									
*I*/(*I*), merged							13.2 (7.9)	13.8 (8.2)	18.4 (10.8)
*R*_int_ § (%)							55.7 (56.1)	54.4 (54.5)	55.7 (55.1)

† Point group 4/*mmm*.

‡ Still data are not scaled by *SADABS* like the rotation data and no error model is determined for . In the merging step is determined from the internal standard deviation 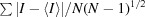.

§ *R*_int_ = 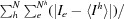 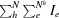, where the summations runs over all *N* unique reflections *h* and equivalents.

**Table 3:** Comparison of stills with *EVAL* rotation data

	*CrystFEL*, Monte Carlo	*EVAL*, Monte Carlo		*EVAL*, Lorentz correction only		*EVAL*, partiality corrected		*EVAL*, partiality corrected, post-refined
	All stills	Consecutive stills	All stills	Consecutive stills	All stills	Consecutive stills	All stills	All stills
*R*_comp_ † (%)	32.0	26.7	26.4	12.9	12.0	6.5	5.3	4.7
*R* on *F* ‡ (%)	18.9	14.5	15.2	10.0	9.0	4.9	4.1	3.1

† Data merged in point group 4/*mmm*.

‡ From *SCALEIT*: overall scale.

**Table 4:** Comparison of data quality

	*EVAL*	*CrystFEL*, Monte Carlo	*EVAL*, Monte Carlo		*EVAL*, Lorentz correction only		*EVAL*, partiality corrected		*EVAL*, partiality corrected, post-refined
	Rotation	All stills	Consecutive stills	All stills	Consecutive stills	All stills	Consecutive stills	All stills	All stils
Refinement†									
*R*_work_ (%)	15.9	17.4	16.8	16.7	16.7	16.6	15.9	16.0	16.0
*R*_free_ (%)	19.8	21.4	20.3	20.2	20.2	20.2	20.0	20.0	20.2
*B* (^2^)	13.8	25.0	20.7	20.6	11.8	11.8	13.0	13.2	13.4
Average anomalous densities‡ ()									
No. of reflections[Table-fn tfn10]	6420	6614	6293	6595	6266	6571	6091	6403	6406
Met SD	13.3	3.2	3.2	4.2	3.6	4.6	5.2	6.3	7.2
Cys SG	10.9	2.1	2.9	3.3	2.8	3.3	3.8	5.2	6.0
Cl	5.0	1.0	1.0	1.0	1.1	1.2	1.7	2.1	2.3
Na^+^	1.8		1.0	0.8	2.0	0.8	2.0	1.5	1.5

† Restraint refinement, individual isotropic *B* factors, input structure PDB entry 193l.

‡ *ANODE* with data merged in point group 422. Averaged densities over similar atom types (two for Met SD, eight for Cys SG, 14 for Cl and one for Na^+^).

§ Selected by *SHELXC*.

**Table 5:** Comparison of data with reduced statistics (60 frames)

	*EVAL*, Monte Carlo		*EVAL*, partiality corrected	
	Consecutive stills	Random stills	Consecutive stills	Random stills
Refinement†				
*R*_work_ (%)	20.7	25.6	17.3	20.7
*R*_free_ (%)	25.1	32.3	21.8	26.3
*B* (^2^)	20.9	21.6	14.3	13.8
Average anomalous densities‡ ()				
No. of reflections§	862	1265	2212	2166
Met SD	0.6	0.8		1.6
Cys SG		0.8	1.1	
Cl				
Na^+^				

† Restraint refinement, individual isotropic *B* factors, input structure PDB entry 193l.

‡ *ANODE* with data merged in point group 422. Averaged densities over similar atom types (two for Met SD, eight for Cys SG, 14 for Cl and one for Na^+^).

§ Selected by *SHELXC*.

## Appendix A Comparison with Laue interference function

The diffracted intensity reflected by a small crystal bathed in an incident monochromatic beam is proportional to the shape transform of the crystal. The reflected intensity received by the detector in a small cone of solid angle dΩ, while the reciprocal-lattice vector  has a small deviation ∊ of the Bragg angle θ, is given by the Laue interference function (Laue, 1936 ▸; James, 1958 ▸) and is used in papers by Kirian *et al.* (2010 ▸) and White *et al.* (2012 ▸),

*N*_1_, *N*_2_ and *N*_3_ are the number of unit cells in the three dimensions of the parallepiped crystal. ξ is the scalar product Δ**k** · **a** and in near-Bragg condition it is equal to *h* + Δ*h*, and likewise for the other directions. As we are only interested in the diffracted intensity close to the Bragg condition, we introduce a local reciprocal axis system and replace ξ by the nonperiodic Δ*h*. The terms in the denominator of (12) can then be written as (πΔ*h*)^2^ because they concern only small numbers,

It is more convenient to choose the reciprocal axes system such that Δ*l* is along the reciprocal-lattice vector  and Δ*h* and Δ*k* are parallel to the diffracting Bragg plane *hkl* (Authier, 2001 ▸). Such a transformation can be carried out because the normal to the Bragg plane is always a reciprocal-lattice vector. (Note that Δ*h*, Δ*k* and Δ*l* are dimensionless.) The Jacobians of the transformation of the integration variables are dΩ = (*d_hkl_*/sinθ)λ^2^d(Δ*h*)d(Δ*k*) and d∊ = λ/(2*d_hkl_*cosθ)d(Δ*l*) (Authier, 2001 ▸), leading to

By integration over Δ*h* and Δ*k* the diffracted intensity for a given value of ∊ is obtained,

The equivalence of this expression to that of James (1958 ▸) and Buerger (1960 ▸), as we use in *EVAL*, is shown by the following. How does Δ*l* depend on a small deviation Δθ = ∊? We write Δ*l* = d(*l*)/dθ = d(*c*/*d*_*hkl*_)/dθ = d(*cd* ^*^ *_hkl_*)/dθ = *c*2cosθ/λ. *cN*_3_ can be written as *ld_hkl_N*_3_, and further we use the property that the volume of the crystal *V*_crystal_= *N*_1_ *N*_2_ *N*_3_ *V*_cell_ and *B* = 2π*d_hkl_*cosθ/λ. Writing (14) in terms of d∊ gives

Using *N*_1_ *N*_2_/*V*_cell_ = *V*_crystal_/*V* ^2^_cell_(1/*N*_3_) (James, 1958 ▸, p. 43) and *d_hkl_* = λ/2sinθ, we can write

(17) is exactly the equation used in *EVAL* (2), as the number of layers *s* = *N*_3_ *l*, and using *n* = *N*_1_ *N*_2_ *N*_3_/*V*_crystal_ we can write *V*_crystal_/*V* ^2^_cell_ = *n* ^2^ *V*_crystal_.
